# Analysis of Omics Data Reveals Nucleotide Excision Repair-Related Genes Signature in Highly-Grade Serous Ovarian Cancer to Predict Prognosis

**DOI:** 10.3389/fcell.2022.874588

**Published:** 2022-06-13

**Authors:** Danian Dai, Qiang Li, Pengfei Zhou, Jianjiang Huang, Hongkai Zhuang, Hongmei Wu, Bo Chen

**Affiliations:** ^1^ Department of Vascular and Plastic Surgery, Guangdong Provincial People’s Hospital and Guangdong Academy of Medical Sciences, Guangzhou, China; ^2^ Department of Cardiology, Guangdong Cardiovascular Institute, Guangdong Provincial People’s Hospital, Guangdong Academy of Medical Sciences, Guangzhou, China; ^3^ Chongqing Key Laboratory of Oral Diseases and Biomedical Sciences, Stomatological Hospital of Chongqing Medical University, Chongqing, China; ^4^ Department of Pathology, Guangdong Provincial People’s Hospital and Guangdong Academy of Medical Sciences, Guangzhou, China; ^5^ Department of General Surgery, Guangdong Provincial People’s Hospital and Guangdong Academy of Medical Sciences, Guangzhou, China; ^6^ Department of Breast Cancer, Guangdong Provincial People’s Hospital and Guangdong Academy of Medical Sciences, Guangzhou, China

**Keywords:** high-grade serous ovarian cancers, nucleotide excision repair, prognosis, nomogram, gene signature predicting prognosis of NER genes

## Abstract

Most of the high-grade serous ovarian cancers (HGSOC) are accompanied by P53 mutations, which are related to the nucleotide excision repair (NER) pathway. This study aims to construct a risk signature based on NER-related genes that could effectively predict the prognosis for advanced patients with HGSOC. In our study, we found that two clusters of HGSOC with significantly different overall survival (OS) were identified by consensus clustering and principal component analysis (PCA). Then, a 7-gene risk signature (DDB2, POLR2D, CCNH, XPC, ERCC2, ERCC4, and RPA2) for OS prediction was developed subsequently based on TCGA cohort, and the risk score-based signature was identified as an independent prognostic indicator for HGSOC. According to the risk score, HGSOC patients were divided into high-risk group and low-risk group, in which the distinct OS and the predictive power were also successfully verified in the GEO validation sets. Then we constructed a nomogram, including the risk signature and clinical-related risk factors (age and treatment response) that predicted an individual’s risk of OS, which can be validated by assessing calibration curves. Furthermore, GSEA showed that the genes in the high-risk group were significantly enriched in cancer-related pathways, such as “MAPK signaling pathway”, “mTOR signaling pathway”, “VEGF signaling pathway” and so on. In conclusion, our study has developed a robust NER-related genes-based molecular signature for prognosis prediction, and the nomogram could be used as a convenient tool for OS evaluation and guidance of therapeutic strategies in advanced patients with HGSOC.

## Introduction

High-grade serous ovarian cancer (HGSOC) is of great concern to the researchers among all ovarian cancers, as it accounts for 70%–80% of deaths from ovarian cancer. The modes of carcinogenesis, molecular-genetic characteristics, and the origin are distinctive from low-grade serous ovarian cancer (Kurman et al., 2014; Bowtell et al., 2015). Due to the lack of effective early screening methods for ovarian cancer, almost 90% of serous ovarian cancer patients are diagnosed as stage III–IV at first diagnosis, while the 10-year survival rate of advanced patients with HGSOC is only 15% (Narod, 2016). So far, surgery remains the most important treatment approach and the subsequent chemotherapy, targeted therapy, radiation, and immunotherapy are used to enhance the curative effect according to the International Federation of Gynecology and Obstetrics (FIGO) staging system (González-Martín et al., 2010; Jessmon et al., 2017). However, for advanced patients, the current FIGO classification method failed to provide accurate information to predict prognosis, nor guide physicians’ treatment decisions (Llueca et al., 2018; Tajik et al., 2018). It is a widespread phenomenon for advanced patients with HGSOC at the same FIGO stage to have completely different prognostic outcomes due to complex biological processes and unintelligible molecular mechanisms. Therefore, it is necessary and urgent to prompt a search for novel and reliable prognostic molecular signatures for predicting prognosis and guiding appropriate therapeutic strategies.

In recent years, genome-wide expression profiling detection can effectively provide detailed information for the prognosis assessment of cancer patients (Anurag et al., 2018; Zhu et al., 2018). In breast cancer, the genomic panel detection methods, which included 21-gene recurrent score and 70-gene MammaPrint assay, could provide some valuable information for the prognosis evaluation and treatment selection of patients (Cardoso et al., 2016; Sparano et al., 2018). Notably, although HGSOC shares high molecular similarity with basal-like breast cancer, there is no molecular evaluation system available for clinical use in HGSOC (Cancer Genome Atlas Network, 2012).

DNA damage response and repair pathways play an essential regulatory role in the occurrence and development of ovarian cancer (Majidinia et al., 2017). DNA repair mechanisms mainly contain various pathways, such as mismatch repair, base excision repair, nucleotide excision repair (NER), homologous recombination, and non-homologous end joining (Mirza-Aghazadeh-Attari et al., 2019). Importantly, HGSOC is mostly accompanied by mutations of P53 that are near related to the NER pathway (Williams and Schumacher, 2016). The NER pathway is composed of various proteins acting in concert and is the main pathway to remove large DNA lesions caused by ionizing radiation and other mutagens (Shuck et al., 2008). In addition, the NER pathway also can repair the damage caused by platinum drugs (such as cisplatin and carboplatin), which are most widely used in the treatment of ovarian cancer. In recent years, a large number of studies have reported that NER-related genes, such as ERCC1, XPC, and GTF2H5, could be used as biomarkers of treatment response or prognosis for tumor patients (Lin et al., 2010; Fleming et al., 2012; Gayarre et al., 2015). Besides, other studies have found that subtle changes of NER function may greatly increase the susceptibility of healthy individuals to lung cancer and head and neck squamous cell cancer (Wei et al., 1996; Cheng et al., 1998). However, despite the increased concern of NER in the field of ovarian cancer, there is still a lack of comprehensive analysis of NER-related genes in advanced patients with HGSOC to assess prognosis and guide therapy strategies effectively.

In the present study, we comprehensively explored the roles of 31 NER-related genes in HGSOC based on multiple transcriptome datasets, such as The Cancer Genome Atlas (TCGA), the Genotype-Tissue Expression (GTEx) Project, and the Gene Expression Omnibus (GEO) database. We evaluated the interaction and correlation among the 31 NER-related genes and employed the consensus cluster analysis to identify two HGSOC clusters with different clinical outcomes based on their expression patterns of these genes. Then we conducted the least absolute shrinkage and selection operator (LASSO) Cox regression to obtain a 7-gene signature on TCGA HGSOC cohort. This robust risk signature was successfully confirmed in two GEO validation sets and showed an excellent predictive effect on prognosis. Moreover, the risk signature and clinical characteristics were used to construct a nomogram to predict the prognosis of advanced patients with HGSOC. Finally, we also used the gene set enrichment analysis (GSEA) to explore the differences in the signaling pathways between subgroups classified by risk signature. Our results indicated that the risk signature derived from seven NER-related genes could serve as novel prognostic biomarkers for advanced patients with HGSOC.

## Materials and Methods

### Public Data Collection and Processing

The study design flowchart is presented in Supplementary Figure S1. The Fragments per Kilobase of transcript per Million mapped reads (FPKM) for RNA-seq data of 378 ovarian cancer tissues and 88 normal ovarian tissues were respectively extracted from two datasets, including the Cancer Genome Atlas (TCGA) and the Genotype-Tissue Expression (GTEx), which were downloaded from UCSC Xena (https://xenabrowser.net/datapages) (Goldman et al., 2018). According to patients’ clinicopathological information of TCGA, RNA-seq data and clinical data of 326 advanced HGSOC samples were eventually enrolled in our analysis. The exclusive criteria were as follows: 1) patients with stage I–II or unclear stage; 2) patients with neoadjuvant chemotherapy; 3) patients with well differentiation or unspecified defined; 4) patients with non-ovarian primary tumor or other histological types; 5) patients with survival less than 30 days. We re-annotated the gene symbols based on the information recorded in the HUGO Gene Nomenclature Committee (HGNC; http://www.genenames.org) (Eyre et al., 2006), and the gene expression of both datasets was unified as log2 (x + 1) to increase comparability. Then, the “*limma*” package was used to average the repeated data of each expression and merge the two datasets with normalization in programming language R (version 4.0.1; https://cran.r-project.org) (Ritchie et al., 2015).

Four independent expression data of advanced patients with HGSOC (GSE13876, GSE49997, GSE17260, and GSE63885) were downloaded from Gene Expression Omnibus (GEO) database (https://www.ncbi.nlm.nih.gov/geo). Gene expression in GSE13876 was performed using Operon human v3 ∼35 K 70-mer two-color oligonucleotide microarrays (GPL7759; N = 415); Gene expression in GSE49997 was performed using ABI Human Genome Survey Microarray Version 2 (GPL2986; N = 204); Gene expression in GSE17260 was performed using Agilent-014850 Whole Human Genome Microarray 4 × 44K G4112F (Probe Name version) (GPL6480; N = 110); Gene expression in GSE63885 was performed using Affymetrix Human Genome U133 Plus 2.0 Array (GPL570; N = 101). Then, we integrated 346 standards-compliant samples from three datasets [GSE49997 (N = 166), GSE17260 (N = 110) and GSE63885 (N = 70)] as a combined validation set based on the exclusive criteria to improve the sample size, and we executed batch normalization between these three platforms using the “*sav*” and “*limma*” packages in R to avoid generating unreliable results (Johnson et al., 2007; Leek et al., 2012). Gene expression values of all GEO datasets were converted by log2 and for genes with multiple probes, and we used average values to represent the performance of specific genes.

### The 31 Candidate Genes

We used the database (TCGA, GTEx, and four GEO databases) for gene screening. A total of 8,466 common genes were selected. According to previous studies (Friedberg, 2001; Marteijn et al., 2014), 31 shared genes among TCGA-GTEx and four GEO datasets related to nucleotide excision repair (NER) were used for our analysis, including RAD23A, RAD23B, RPA2, RPA3, ERCC8, POLR2A, POLR2B, POLR2C, POLR2D, POLR2F, POLR2G, POLR2K, POLR2L, DDB1, DDB2, LIG1, GTF2H1, GTF2H3, GTF2H5, CUL3, CUL4A, CUL5, RBX1, CCNH, CDK7, XPA, XPC, ERCC2, ERCC3, ERCC4, and MNAT1.

### mRNA Expression Analysis of 31 NER-Related Genes

We first analyzed the mRNA expression levels between HGSOC tissues and normal tissues by using the “limma” package with cut-off criteria of *p* < 0.05. The mRNA expression profiles of 31 NER-related genes were obtained. The heatmap and violin plots were presented by “*pheatmap*” and “*ggplot2*” packages in R.

### Protein-Protein Interaction Network Analysis and Correlation Analysis

All 31 NER-related genes were used for the protein-protein interaction (PPI) analysis with a combined confidence score  ≥ 0.9 *via* the Search Tool for the Retrieval of Interacting Genes (STRING) (https://string-db.org/) (Szklarczyk et al., 2015). Pearson correlation analysis was utilized to present the collinearity among different NER-related genes.

### Consensus Clustering Analysis and Principal Component Analysis

The “*ConsensusClusterPlus*” package in R (50 iterations, resample rate of 80%) was applied to explore the clinical implications of the 31 NER-related genes in the TCGA HGSOC cohort. The number of clusters and their stability were determined by the consensus clustering algorithm (Kanungo et al., 2002). Then, the TCGA HGSOC cohort was clustered into different two clusters. To further confirm the rationality of clustering, principal component analysis (PCA) was carried out in R to observe the distribution of gene expression in two clusters. The Kaplan-Meier method and log-rank test were used to present the difference of overall survival (OS) between two clusters. The associations between the clinical characteristics and two different clusters were analyzed by Chi-square test.

### Construction of Gene Signature

Seven candidate genes associated with OS (*p* < 0.1) were determined by the univariate Cox regression analysis of 31 NER-related genes; the hazard ratios (HRs) of genes <1 or >1 were regarded as protective or risk genes, respectively. To prevent over-fitting in our analysis, the least absolute shrinkage and selection operator (LASSO) regression analysis was used to identify the optimal prognostic model out of the selected seven candidate genes (Goeman, 2010), and genes’ coefficients were determined based on the best penalty parameter λ. The risk score for the signature was estimated accurately using the following formula:

$$\text{Risk}\;\text{Score}=\sum_{i=1}^{n}\text{Coefi}*\text{Expi}$$

N is the number of selected genes, Coefi is the regression coefficient generated by the LASSO regression and Expi is the expression value of each selected gene. TCGA HGSOC patients were divided into low- and high-risk groups according to the median risk score. Kaplan-Meier (K-M) survival was used to estimate the OS between two risk groups. In addition, the receiver operating characteristic (ROC) curves and area under the ROC curves (AUC) were also performed to estimate sensitivity and specificity. Chi-square test was used to assess the association between the clinical characteristics and two risk groups.

### Validation of the Prognostic Signature

Similarly, the risk score of each advanced patient with HGSOC from the GSE13876 and the merged GEO datasets was calculated based on the formula above. Taking the median risk score in the TCGA cohort as the cut-off value, HGSOC patients in both validation sets were divided into high- or low- groups. The K-M method and log-rank test were employed to calculate OS with an overall significance level of *p* < 0.05. The risk score was identified as an independent prognostic factor by the method of the univariate and multivariate Cox regression analyses. Moreover, the prognostic nomogram based on the risk score of NER-related signature and clinical-related variables was constructed. The performance of the prediction model developed was validated by assessing the internal calibration curves of the TCGA cohort. To further confirm the expression of seven selected genes, we analyzed normal ovarian tissues and serous ovarian cancer tissues obtained from the Human Protein Atlas (HPA) database.

### Gene Set Enrichment Analysis

Gene set enrichment analysis (GSEA) was used in the TCGA cohort to investigate the potential biological pathways underlying the different risk groups defined by the 7-gene expression signature. Kyoto Encyclopedia of Genes and Genomes (KEGG) gene sets (v7.1) and phenotype label (high risk vs. low risk) files were generated and loaded into the GSEA software (v4.0.3; Broad Institute, Cambridge, MA). The permutation test run 1,000 times. The pathways with normalized enrichment score (NSE) absolute value >1, normalized *p*-value < 0.05, and false discovery rate (FDR) q-value < 0.25 were significantly enriched.

### Statistical Analysis

Unless otherwise specified, all statistics analyses were performed with R software. The differences between groups for the continuous and categorical variables were respectively assessed by studentʼs t-test or one-way ANOVA and the Chi-square test. The “*limma*”, “*sva*”, “*ConsensusClusterPlus*”, “*pheatmap*”, “*ggplot2*”, “corrplot”, “*pROC*”, “*rms*”, “*survival*” packages were used for analysis or visualization in R. All statistical tests were two-sided and *p* < 0.05 was considered statistically significant.

## Results

### The Expression of 31 NER-Related Genes Between Tumor Samples and Normal Control Samples

**FIGURE 1:**
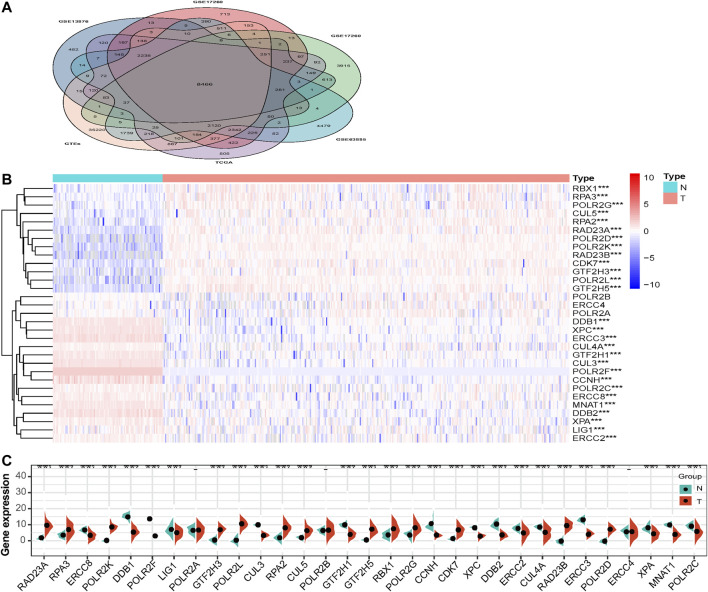
The expression levels of NER-related genes between tumor samples and normal samples in TCGA HGSOC cohort and GTEx normal ovary cohort. **(A)** Venn diagram displays that 8,466 shared genes were contained in the TCGA, GTEx, and four GEO datasets. **(B)** The TCGA and GTEx databases were used to jointly analyze 31 NER-related genes and the heatmap was used to visualize the expression levels of these genes in each clinical sample. **(C)** The violin-plot shows the expression of 31 NER-related genes between tumor tissues and the normal tissues. ∗*p* < 0.05, ∗∗*p* < 0.01, and ∗∗∗*p* < 0.001. Abbreviation: HGSOC: high-grade serous ovarian cancer; TCGA: The Cancer Genome Atlas; GTEx: the Genotype-Tissue Expression project; GEO: Gene Expression Omnibus; NER: Nucleotide excision repair.

To better understand the importance of the NER-related genes in tumor initiation and progression, we firstly investigated expression levels of NER-related genes in different tissue samples of the merged TCGA-GTEx dataset. The TCGA-GTEx cohort comprised 326 advanced patients with HGSOC and 88 ovaries of healthy donors. The clinicopathological characteristics of the TCGA HGSOC cohort were listed in Table 1. We selected 31 NER-related candidate genes among 8,466 shared genes in all datasets for further analysis (Figure 1A). Then, mRNA expression levels of the 31 genes were presented for HGSOC along with corresponding normal controls by the heatmap (Figure 1B). Overall, the expression levels of 13 NER-related genes were significantly increased, and 15 genes were significantly downregulated in HGSOC tissues when compared to those with normal controls (Figure 1C). However, the expression levels of three genes (including POLR2B, ERCC4, and POLR2A) had no significant difference.

**TABLE 1:** The clinical characteristics of the HGSOV cohort in the TCGA database.

Variable	Number
Age	
Mean (SD)	59.29 (11.30)
Pathological diagnosis	
Serous	326
Grade	
G2	34 (10.4%)
G3	292 (89.6%)
FIGO stage	
IIIA	6 (1.8%)
IIIB	13 (4.0%)
IIIC	256 (78.5%)
IV	51 (15.6%)
Treatment response	
CR	183 (56.1)
PR	42 (12.9%)
SD	20 (6.1%)
PD	24 (7.4%)
Unknown	57 (17.5%)
Residual tumor (post-operation)	
<1 cm	211 (64.7%)
≥1 cm	87 (26.7%)
Unknown	28 (8.6%)

Abbreviation: HGSOV, High-grade serous ovarian cancers; TCGA, The Cancer Genome Atlas; SD, Standard deviation; G2, Moderately differentiated; G3, Poorly differentiated; FIGO, International Federation of Gynecology and Obstetrics; CR, Complete remission; PR, Partial remission; SD, Stable disease; PD, Progressive disease

### The Interaction and Correlation Among the NER-Related Genes

**FIGURE 2:**
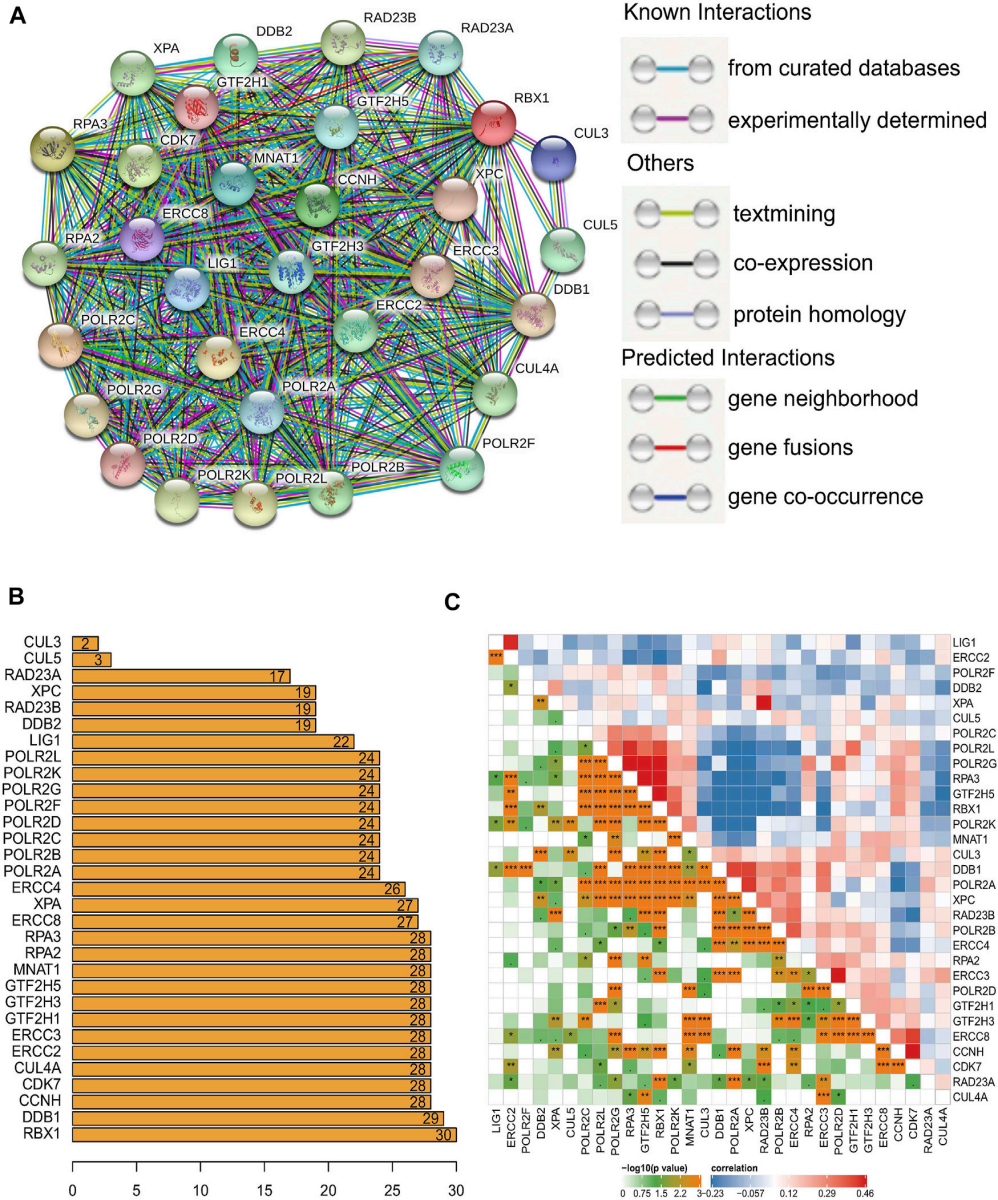
The interaction and correlation among 31 NER-related genes. **(A)** The PPI network of the 31 NER-related genes was constructed by STRING database and each line represents a reported physical protein–protein interaction between nodes. **(B)** The number of bar graphs represents the total connections of each node to other nodes frequency. **(C)** The Pearson correlation analysis shows the collinearity among 31 NER-related genes. Abbreviation: NER: Nucleotide excision repair; PPI: Protein-protein interaction; STRING: the Search Tool for the Retrieval of Interacting Genes.

The interaction relationships among the 31 NER-related genes were shown by the PPI network, and the number of interactions for each gene was counted in Figure 2. Our results showed that the interrelationships among 31 genes were of high closeness and great complexity (Figure 2A). Except CUL3 and CUL5, the other 29 genes seemed to be the hub genes of the interaction network, because they had interactions with more than half of the genes (Figure 2B). We also conducted correlation analysis and observed that there were various degrees of positive and negative collinearity among some NER-related genes in HGSOC (Figure 2C). We believe that the changes in the correlation of 31 NER-related genes may reflect the inherent characteristics of antagonistic or synergistic effects between the corresponding transcribed functional proteins.

### Consensus Clustering of NER-Related Genes Identified Two Clusters of HGSOC With Different Prognostic Outcomes

**FIGURE 3:**
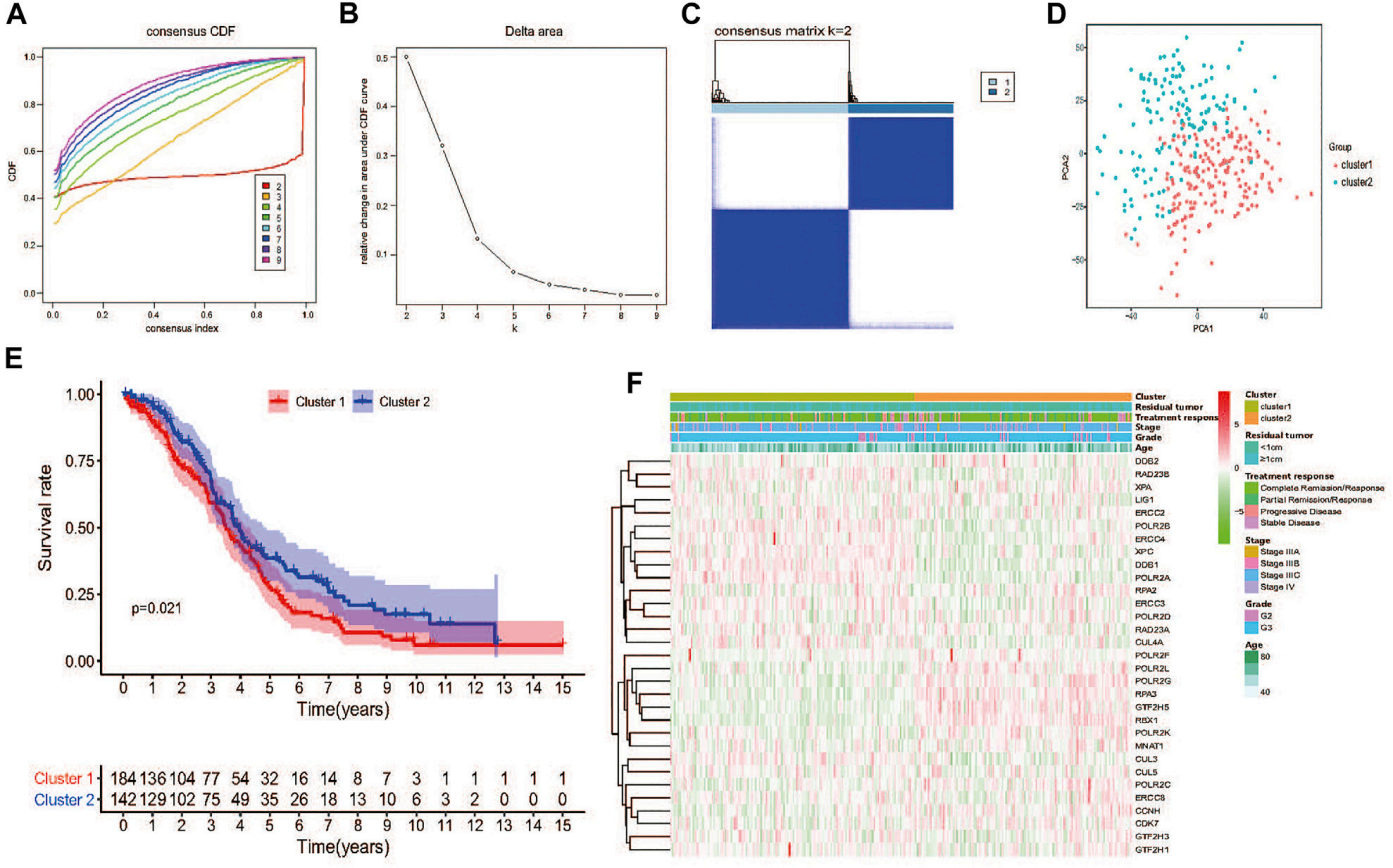
Consensus clustering and overall survival of TCGA HGSOC patients in the different two clusters. **(A)** Consensus clustering CDF for k = 2 to 9. **(B)** Relative change in area under CDF curve for k = 2 to 9. **(C)** The TCGA HGSOC patients was divided into two distinct clusters when k = 2. Consensus clusteringmatrix for k = 2. **(D)** PCA of the total mRNA expression profile in the TCGA dataset. HGSOC patients in the cluster1 subgroup are marked with red, HGSOC patients in cluster2 are marked with green. **(E)** Kaplan–Meier OS curves for different clusters. **(F)** No significant difference was found for the clinicopathologic features between cluster1 and cluster2.

Next, TCGA HGSOC samples were selected for the subsequent consensus clustering analysis. According to the expression similarity of the 31 NER-related genes, k = 2 could be the optimal choice when clustering stability datasets increased from k = 2–9 (Figures 3A–C). We noticed that the distribution of sample numbers in each group was roughly balanced, and the interference between groups was minimal in the two groups when k = 2 (Figure 3C). Hence, TCGA HGSOC samples were correspondingly classified as two groups (184 samples in cluster1 and 142 samples in cluster2) through consensus cluster analysis. The clinical characteristics of the two clusters were shown in Supplementary Table S1. Moreover, Principal component analysis (PCA) was used to compare the difference of transcriptional profiles between the two clusters, and the results exhibited a significant distinction (Figure 3D). Besides, advanced patients with HGSOC were found to have the significantly lower OS in the cluster1 than those in the cluster2 (*p* = 0.021), which suggested that the 31 NER-related genes could classify the advanced patients with HGSOC at the prognostic level (Figure 3E). We then analyzed the associations between the clusters and clinicopathological characteristics. However, no significant difference was found between two clusters in the age, grade, stage, treatment response, and residual tumor (all *p* > 0.05).

### Identification of Prognostic Value and a Risk Signature Based on NER-Related Genes

**FIGURE 4:**
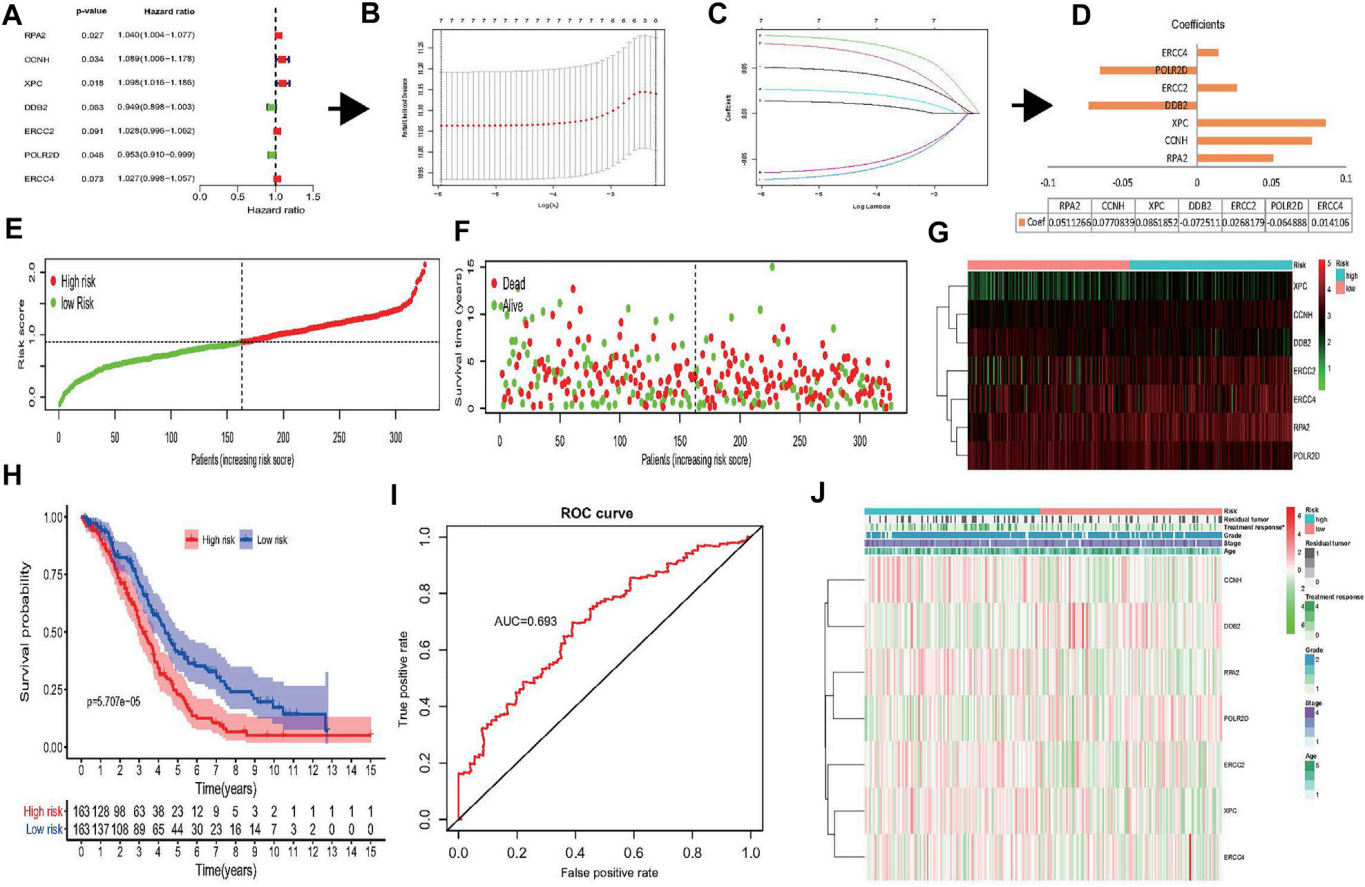
Construction of risk signature based on seven NER-related genes. **(A–D)** The process of constructing the signature based on seven NER-related genes. **(A)** Univariate Cox regression analysis of the NER-related genes was identified seven genes that potentially correlated with OS (*p* < 0.1). **(B)** LASSO algorithms was used to identify and evaluate the 7-gene signature in the TCGA HGSOC cohort and seven genes were finally selected and used to develop a risk signature to predict patients prognosis. **(C)** LASSO coefficient profiles of the seven genes based on the TCGA HGSOC cohort. **(D)** The coefficients estimated by multivariate Cox regression via LASSO are presented. **(E–G)** Visualization of the association of the risk scores with survival status and gene expression profiles in HGSOC. **(H)** The OS was remarkably worse in the high-risk group than that of low-risk group. **(I)** ROC curve was used to evaluate the prediction efficiency of the risk signature. **(J)** Significant differences were found for the treatment response between high- and low-risk groups. Abbreviation: NER: Nucleotide excision repair; OS: Overall survival; LASSO: Least absolute shrinkage and selection operator; TCGA: The Cancer Genome Atlas; HGSOC: Highly-grade serous ovarian cancer; ROC: Receiver operating characteristic.

To further explore the prognostic value of 31 NER-related genes, univariate Cox regression analysis was performed based on the mRNA expression levels of genes from TCGA. The results demonstrated that seven out of the 13 NER-related genes were potentially associated with the OS (*p* < 0.1). Among these seven genes, only DDB2 and POLR2D were considered as protective genes with HR <1, while RPA2, CCNH, XPC, ERCC2, and ERCC4 were considered as risky genes with HR >1 (Figure 4A).

According to our previous results, the collinearity between these genes may affect the accuracy of traditional Cox regression analysis (Figure 2C). Therefore, the LASSO Cox regression method to the seven potentially prognosis-related genes was performed to identify the most powerfully prognostic NER-related genes finally. The LASSO results demonstrated that all seven genes were chosen to construct the prognostic risk signature (Figures 4B,C), and the coefficients of selected genes were shown in Figure 4D. Then, the risk scores were calculated according to the coefficients, and the median risk score was the cut-off value. A total of 326 HGSOC patients were divided evenly into the high- and low-risk groups. The distributions of the 7-gene signature-based risk scores, OS status, and mRNA expression profiles were displayed in Figures 4E–G. The K-M survival plot showed that the OS of the advanced patients with HGSOC was significantly worse in the high-risk group than that in the low-risk group (*p* = 5.707e-05) (Figure 4H). The 5-year OS was 14.1% in high-risk group and 27.0% in low-risk group. The predicting power of the risk signature showed well-prediction efficiency with the AUC value equal to 0.693 (Figure 4I). Next, the associations between the risk groups and clinicopathological characteristics were also investigated in the present study (Figure 4J and Supplementary Table S2). The results showed that except treatment response (*p* = 0.049), there were no significant differences between the high- and low-risk groups in the age, grade, stage, and residual tumor (all *p* > 0.05).

**FIGURE 5:**
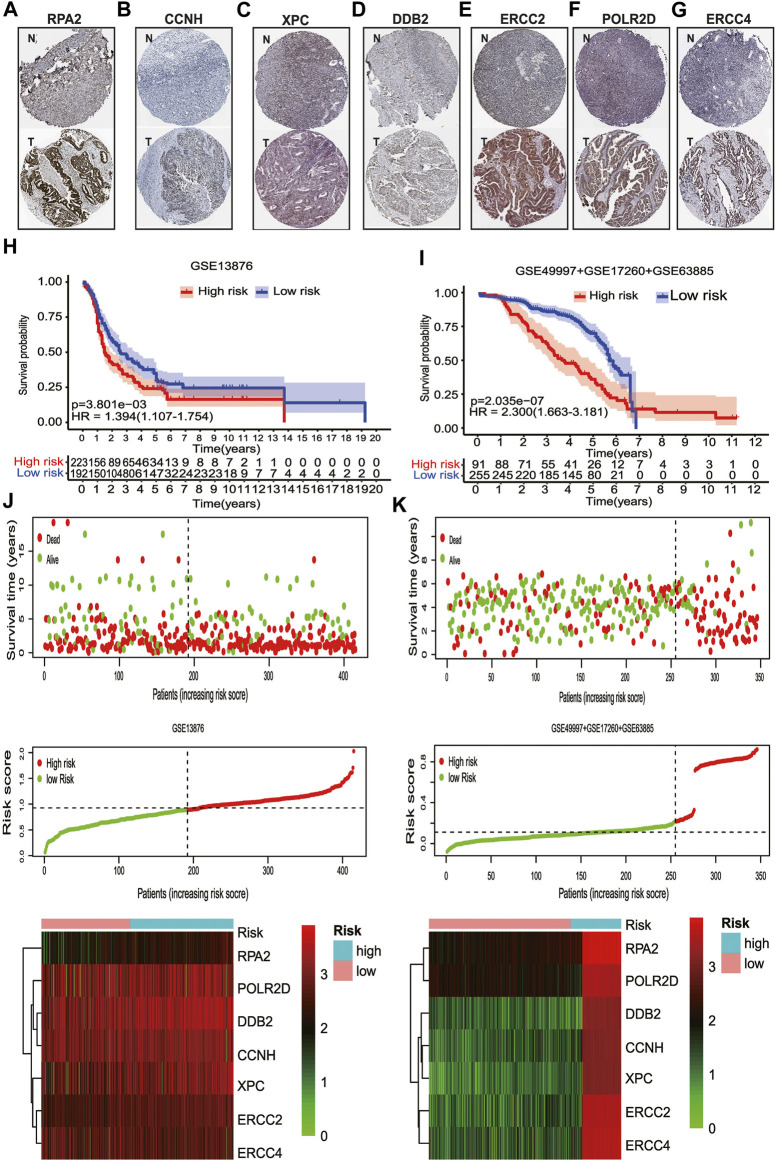
The protein expression levels of seven NER-related genes by IHC staining and the validation of the prognostic signature. **(A)** Representative IHC staining of RPA2 in serous ovarian cancer and normal ovarian tissues. **(B)** Representative IHC staining of CCNH in serous ovarian cancer and normal ovarian tissues. **(C)** Representative IHC staining of XPC in serous ovarian cancer and normal ovarian tissues. **(D)** Representative IHC staining of DDB2 in serous ovarian cancer and normal ovarian tissues. **(E)** Representative IHC staining of ERCC2 in serous ovarian cancer and normal ovarian tissues. **(F)** Representative IHC staining of POLR2D in serous ovarian cancer and normal ovarian tissues. **(G)** Representative IHC staining of ERCC4 in serous ovarian cancer and normal ovarian tissues. **(H)** For advanced patients with HGSOC, the high-risk group had a significantly worse OS than that in the low-risk group in GSE13876. **(I)** For advanced patients with HGSOC, the high-risk group had a significantly worse OS than that in the low-risk group for the combined GEO datasets. **(J)** Visualization of the association of the risk scores with survival status and gene expression profiles in GSE13876. **(K)** Visualization of the association of the risk scores with survival status and gene expression profiles in the combined GEO datasets. Abbreviation: T: Serous ovarian cancer; N: Normal ovarian tissue; NER: Nucleotide excision repair; HPA: The human protein atlas; IHC: Immunohistochemistry. GEO: Gene Expression Omnibus; HGSOC: Highly-grade serous ovarian cancer; OS: Overall survival.

Moreover, the Human Protein Atlas (HPA) database was used to validate the cellular sub-localization and expression patterns of the seven selected genes in serous ovarian cancer tissues and normal ovarian tissues at the protein levels (Figures 5A–G). HPA analysis showed that at protein levels, the expressions of DDB2, POLR2D, CCNH, and RPA2 in HGSOC tissues and normal controls were similar to the mRNA level changes and were mainly located in the nucleus (Figures 1C, 5). However, XPC and ERCC2 did not show significant distinctions at the protein levels between serous ovarian tissues and normal ovarian tissues (Figures 5C,E). The heterogeneity between the HPA data and TCGA RNA-seq data may be ascribed to the differences in post-transcriptional regulation. Altogether, the results further verified that the regulation of NER-related genes was highly disordered in serous ovarian cancer.

### Validation of the Risk Signature to Predict OS of Advanced Patients With HGSOC

**FIGURE 6:**
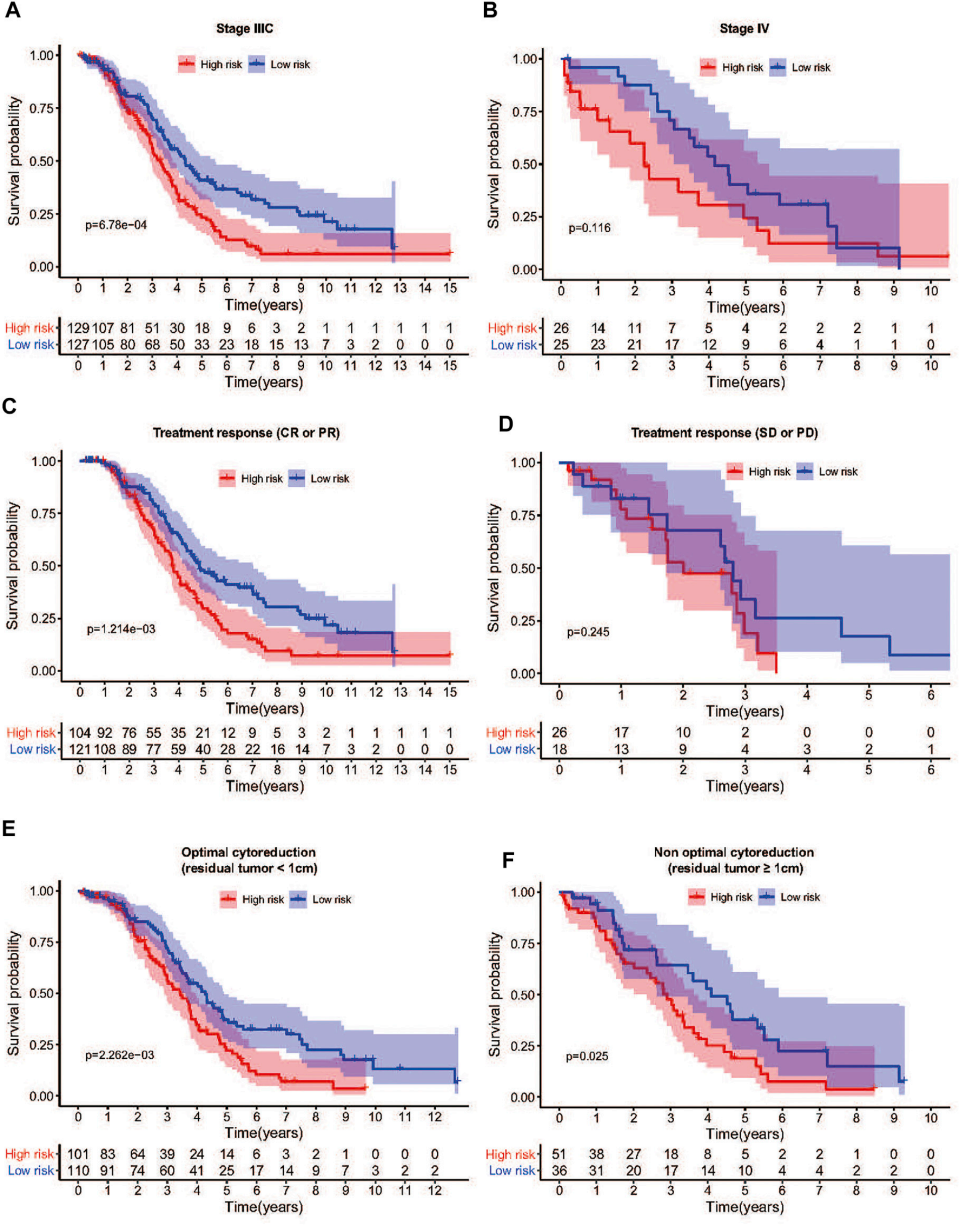
Differences in survival between high- and low-risk groups stratified by clinicopathological factors in TCGA cohort. **(A)** The OS was remarkably worse in the high-risk group than that of low-risk group in FIGO stage IIIC patients. **(B)** The low-risk group showed a better prognosis trend than the high-risk group in FIGO stage IV patients. **(C)** Among patients who achieved CR or PR after treatment, the OS of the high-risk group was also shorter than that of the low-risk group. **(D)** Among patients who achieved SD or PD after treatment, there was no significant difference in OS between the high-risk group and the low-risk group. **(E)** The OS was remarkably worse in the high-risk group than that of low-risk group among patients with optimal cytoreductive surgery. **(F)** The OS was remarkably worse in the high-risk group than that of low-risk group among patients with non-optimal cytoreductive surgery. Abbreviation: OS: Overall survival; CR: Complete remission; PR: Partial remission; SD: Stable disease; PD: Progressive disease.

To confirm that the NER-related genes based on classifier had similar prognostic value in different cohorts, we assessed the samples in GSE13876 (*n* = 415) and the merged GEO dataset (*n* = 346), respectively. According to the median risk score as the cut-off value in the TCGA HGSOC cohort, 192 (46.3%) patients were classified as low-risk, and 223 (53.7%) as high-risk in GSE13876; 255 (73.7%) patients were classified as low-risk, and 91 (26.3%) as high-risk in the merged GEO datasets. The corresponding 5-year OS was 24.1% for the high-risk group and 35.0% for the low-risk group in GSE13876 (HR: 1.394, 95% CI: 1.107–1.754; *p* = 3.901e-03; Figure 5H). Similarly, in the merged GEO validation set, the 5-year OS was 35.3% for the high-risk group and 67.8% for the low-risk group (HR: 2.300, 95%CI: 1.663–3.181; *p* = 2.035e-07; Figure 5I). The distributions of the risk scores, OS status, and mRNA expression profiles of the two validation sets were respectively conducted (Figures 5J,K). As shown in Figures 6A–F, the worse OS rate was observed in the high-risk group compared to that in the low-risk group for patients with FIGO stage IIIC (*p* = 6.78e-04), or patients with CR/PR (*p* = 1.214e-03), or patients with optimal cytoreductive surgery (*p* = 2.263e-03), or those with non-optimal cytoreductive surgery (*p* = 0.025). However, no significant difference was observed for OS between high- and low-risk groups for patients with FIGO stage IV (*p* = 0.116), or patients with SD/PD (*p* = 0.245) due to sample size limitation.

### Nomogram Combined NER-Related Risk Signature and Clinical-Related Features to Predict Patients’ OS

**FIGURE 7:**
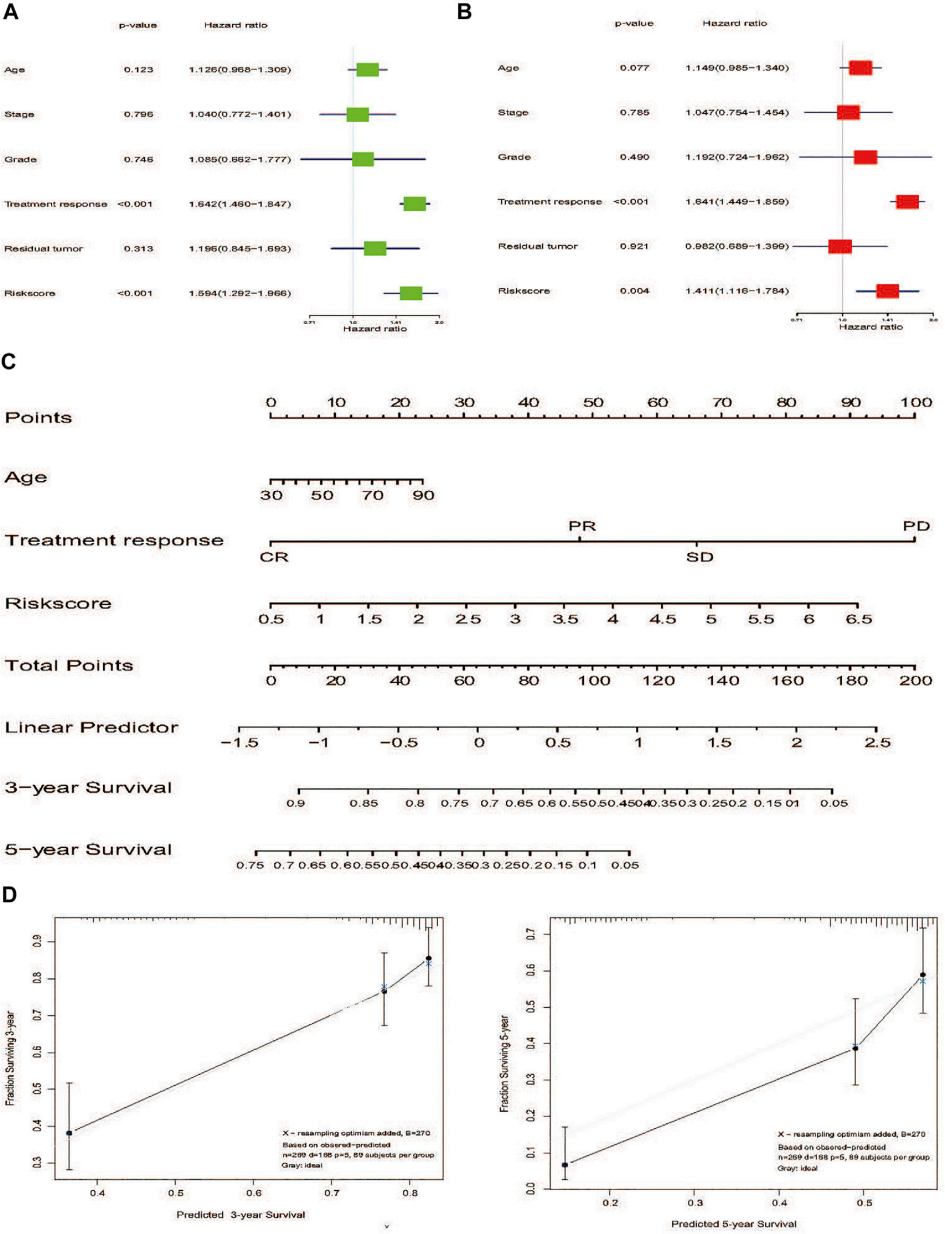
Nomogram development of 7-gene signature to predict the risk of survial in advanced patients with HGSOC. **(A)** Univariate Cox regression analysis of the risk score and clinicopathological factors to identify the indicators that were significantly associated with OS. **(B)** Multivariate Cox regression analysis of the risk score and clinicopathological parameters to reveal the independent prognostic factors. **(C)** A nomogram was constructed to predict three- or 5-year survival. **(D)** Calibration curves of the nomogram to predict 3-or 5-year OS in TCGA internal validation. The actual OS is plotted on the y-axis; nomogram predicted probability is plotted on the x-axis. Abbreviation: NER: Nucleotide excision repair; HGSOC: Highly-grade serous ovarian cancer; OS: Overall survival.

To investigate whether the prediction was better by the risk signature constructed from mRNA expression than from any other clinical-related features, various variables, such as age, stage, grade, treatment response, and residual tumor were included as the potential prognostic factors. In the TCGA HGSOC cohort, the results of both univariate and multivariate analysis revealed that treatment response and risk signature were significantly associated with OS (Figures 7A,B). Due to the potential impact of age on OS (*p* = 0.077), a nomogram that combined the age, treatment response, and risk signature was developed to predict 3- or 5-year survival of advanced patients with HGSOC (Figure 7C). The calibration plots for the nomogram presented the acceptability and conformance in the original cohort between the nomogram forecast and actual observation for the 3- or 5-year OS (Figure 7D). Conclusively, we constructed a nomogram combining various clinical-related factors. NER-related risk signature could provide the most useful and accurate information for the prognosis of these advanced patients with HGSOC.

### Gene Set Enrichment Analysis of High-Risk and Low-Risk Groups

To better understand the significance of risk signature based NER-related genes, GSEA analysis was performed to scrutinize the signaling pathways between the high-risk group and low-risk group from the TCGA cohort. Intriguingly, genes involved in the following biological processes/signaling pathways showed active expression in the high-risk group: mTOR signaling pathway (NSE = 2.15, normalized *p* < 0.0001, FDR q = 0.009), inositol phosphate metabolism (NSE = 2.14, normalized *p* < 0.0001, FDR q = 0.007), phosphatidylinositol signaling system (NSE = 2.13, normalized *p* < 0.0001, FDR q = 0.006), MAPK signaling pathway (NSE = 1.97, normalized *p* < 0.0001, FDR q = 0.014), and VEGF signaling pathway (NSE = 1.94, normalized *p* < 0.0001, FDR q = 0.018). In contrast, two downregulated biological processes/signaling pathways were observed in the high-risk group: DNA replication (NSE = -1.82, normalized *p* = 0.024, FDR q = 0.144) and spliceosome (NSE = -1.80, normalized *p* = 0.037, FDR q = 0.112) (Supplementary Figure S2). These results above showed the risk signature identified based on the seven NER-related genes were closely associated with the malignancy of HGSOC.

## Discussion

Serous ovarian cancer was divided into low-grade serous ovarian cancer and HGSOC, and they had significantly different features in genomics, clinical manifestations, origin, and prognosis (Schmeler and Gershenson, 2008). The previous study initially explored the value of a single NER-related gene in HGSOC (Gayarre et al., 2015), but we considered that it failed to provide a robust predictive efficacy due to the expression of a single gene restricted by multiple factors. Therefore, we comprehensively analyzed the specific value of 31 NER-related genes in HGSOC. Our results demonstrated that the expression levels of NER-related genes were closely associated with the prognostic outcomes of advanced patients with HGSOC. Firstly, we compared the expression levels of 31 NER-related genes using the merged TCGA-GTEx dataset and found that most genes were abnormally expressed in HGSOC. Then, two clusters (cluster1 and cluster2) of HGSOC with completely different prognosis were identified through consensus clustering. Also, based on the data of TCGA HGSOC, a prognostic risk signature of seven NER-related genes was retrieved through the LASSO algorithm. Advanced patients with HGSOC were assigned into two risk subgroups with significant differences for OS according to the risk signature. More importantly, in the external validation datasets, the risk signature was further successfully confirmed as a highly robust prognostic indicator. In addition, a stratified analysis based on clinical factors demonstrated the robustness of the risk signature in prognostic evaluation, and it also implied that the difference in survival based on the risk signature was more likely to be associated with the tumor inherent biological characteristics. Taking advantage of the risk signature, we constructed a nomogram including 7-gene signature and clinical-related factors (age and treatment response) to predict OS of advanced HGSOC patients. Finally, the GSEA analysis explored the differences in oncology-related pathways and key biological processes between the two risk subgroups classified based on the risk signature. Overall, our nomogram could be used as a prognostic classification tool and help clinicians make individualized therapeutic strategies for advanced patients with HGSOC.

NER is a complex biochemical process that requires multiple proteins assemble in an ordered at base damaged sites and then function as a multi-protein complex (De Boer and Hoeijmakers, 2000; Hoeijmakers, 2001). In our research, we also noticed the complex association between various genes expression levels and protein interactions in the NER pathway of HGSOC patients (Figure 2). Surprisingly, two clusters with distinctive OS were identified based on 31 NER-related genes consensus clustering analysis (Figure 3), which indicated the practical possibility of further distinguishing the HGSOC patients at the molecular level.

We also identified a risk signature of seven genes consisting of ERCC2, ERCC4, POLR2D, DDB2, XPC, CCNH, and RPA2 that predicts OS in the TCGA and GEO datasets. ERCC2 single nucleotide polymorphisms (SNPs) were found to be associated with an increased risk of ovarian cancer (Bernard-Gallon et al., 2008; Bicher et al., 1997). The same is applied for ERCC4 (Osorio et al., 2013). POLR2D is also known as DNA-directed RNA polymerase II, which was associated with shorter disease-free survival in prostate cancer (Yamada et al., 2018). In contrast, our results showed that POLR2D was a protective factor for the OS in HGSOC patients (Figure 4A), and this difference may attribute to the fact that the same gene may play different roles in the occurrence and development of different cancers. The other protective factor discovered in our study was DDB2 (Figure 4A), and it was reported that DDB2 could repress ovarian cancer stem cell properties (Cui et al., 2018). In addition, the highly expressed DDB2 could enhance the sensitivity of ovarian cancer cells to cisplatin by increasing cell apoptosis (Barakat et al., 2010). Moreover, downregulation of XPC could also enhance the sensitivity of ovarian cancer to cisplatin (Zhang et al., 2015), and XPC SNPs were correlated with survival outcomes of ovarian cancer treated with platinum-based chemotherapy (Kang et al., 2013). Our results also demonstrated that the low-risk group was associated with better treatment response, which meant that these patients had upregulated DDB2 and downregulated XPC expression and were more sensitive to chemotherapy (Figure 4J). In addition, CCNH was related to the promotion of cancer cell migration (Wang et al., 2013), and RPA2 expression was an independent predictor of adverse outcome in ovarian cancer (Levidou et al., 2012). Taken together, these findings revealed the universal importance of NER-related genes, and indicated that these inhibitor or promotor genes in cancer development were interdependent.

The mechanisms were further investigated to reveal the causes of the different prognosis of the two HGSOC risk subgroups stratified by the 7-gene signature. GSEA demonstrated that some pivotal signaling pathways and biological processes were significantly enriched in the high-risk group with poor survival, including inositol phosphate metabolism, MAPK signaling pathway, mTOR signaling pathway, phosphatidylinositol signaling system, and VEGF signaling pathway. It was reported that inositol phosphate recycling regulated glycolytic and lipid metabolism that drove cancer aggressiveness (Benjamin et al., 2014). It was known that MAPK signaling pathway, mTOR signaling pathway, and VEGF signaling pathway were crucial in tumorigenesis, progression, and drug therapy (Pópulo et al., 2012; Santarpia et al., 2012; Apte et al., 2019). In the low-risk group, GSEA enriched two significant biological processes related to cancer, including DNA replication and splicesome (Herrick and Bensimon, 2009; Ladd et al., 2013). In the current study, the cellular process of NER-related DNA repair was consistent with the biological function of these identified signal pathways. DNA damage and repair processes affect most of all aspects of biological processes, including RNA metabolism, protein translation, and modification. Therefore, the different clinical phenotypes (high-risk group and low-risk group) were further supported by the difference in signal pathways and biological processes.

Despite encouraging findings in the present study, several limitations still exist. First of all, our results are mainly based on bioinformatics analysis. Although there are multiple datasets for mutual verification, experimental and clinical data will be needed to verify our results in the future. Secondly, although the risk signature and nomogram showed good prediction accuracy in the internal verification, their performance is still warranted validation in different HGSOC populations. Finally, our study did not contain clinicopathological information such as the scope of surgical resection and specific chemotherapy drugs, since TCGA did not cover such information, and the treatment standard for advanced patients with HGSOC has been controversial.

## Conclusion

Taken together, we profiled the sharply altered NER-related genes between HGSOC and normal samples, which may play a vital role in the progression of HGSOC. More importantly, a robust risk signature that was significantly associated with the clinical outcome of HGSOC was constructed and validated in two different GEO validation sets. In addition, we also developed a 7-gene nomogram containing the risk signature and clinical-related risk factors, which may aid the individualized prediction of the prognosis of advanced patients with HGSOC. Finally, further research on these genes may provide new insights into the potential relationship between the NER repair pathway and HGSOC prognosis.

## Data Availability

The datasets presented in this study can be found in online repositories. The names of the repository/repositories and accession number(s) can be found in the article/Supplementary Material.

## Supplementary Material

Supplementary Figure S1 The flow chart of the study design and analysis.

Supplementary Figure S2 Differential signaling pathways and cellular processes between risk subgroups defined by 7 NER-related gene signatures.
