# Supplementary figures and images for: Analysis of Omics Data Reveals Nucleotide Excision Repair-Related Genes Signature in Highly-Grade Serous Ovarian Cancer to Predict Prognosis
Source: Front Cell Dev Biol. 2022 Jun 13;10:874588. doi: 10.3389/fcell.2022.874588 (PMC9235032; doi:10.3389/fcell.2022.874588)

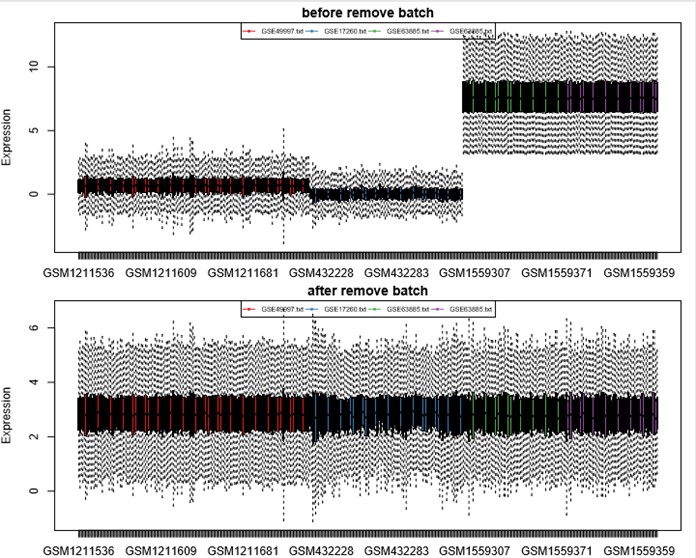

Supplement: Supplementary file 1 [file Image3.JPEG]

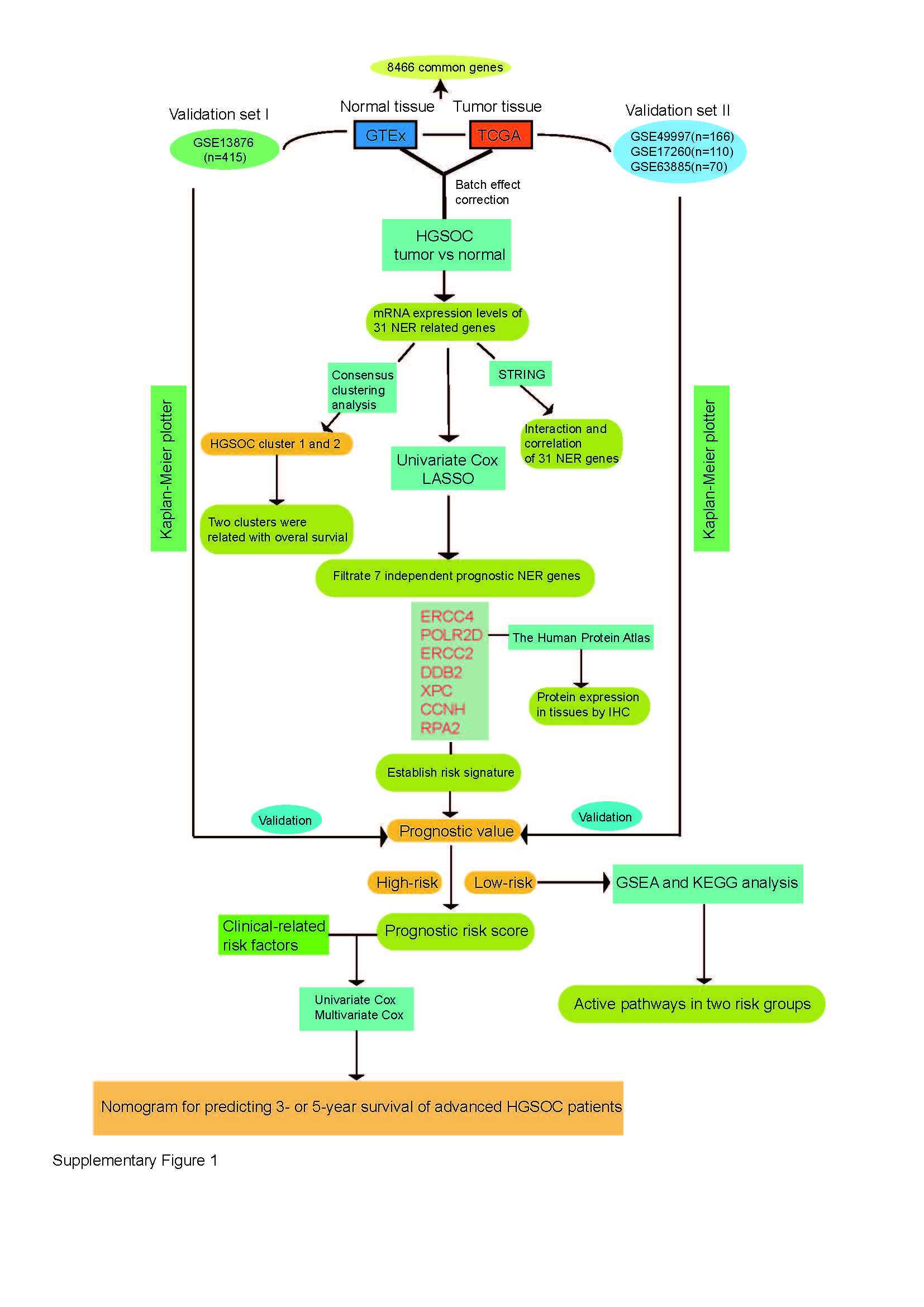

Supplement: Supplementary file 2 [file Image1.JPEG]

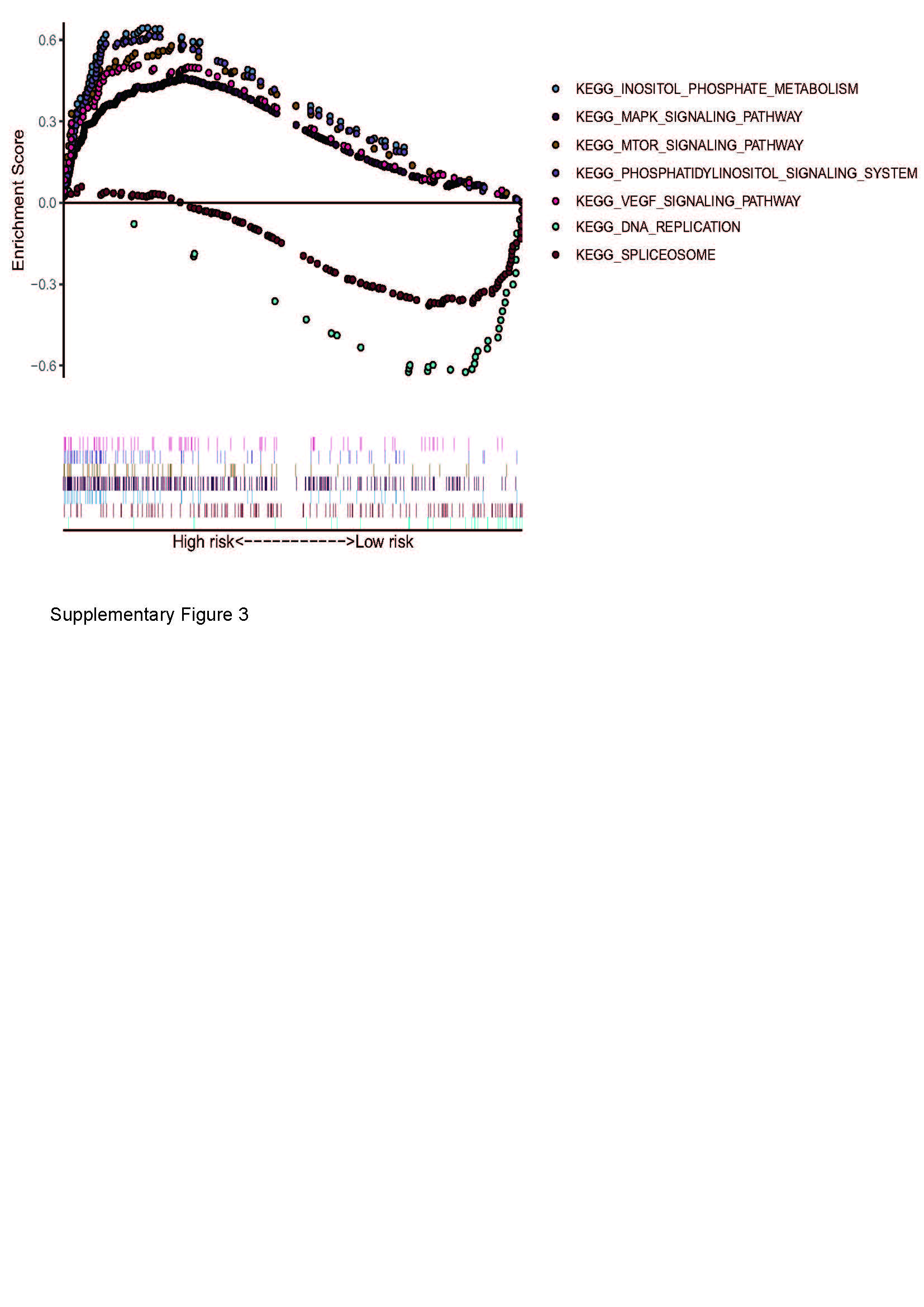

Supplement: Supplementary file 3 [file Image2.JPEG]
