# Supplementary material for: Analysis of Omics Data Reveals Nucleotide Excision Repair-Related Genes Signature in Highly-Grade Serous Ovarian Cancer to Predict Prognosis
Source: Front Cell Dev Biol. 2022 Jun 13;10:874588. doi: 10.3389/fcell.2022.874588 (PMC9235032; doi:10.3389/fcell.2022.874588)
Supplement: Supplementary file 4 [file DataSheet1.docx]

| Table S1. Clinicopathological characteristics of advanced patients with HGSOC between cluster1 and cluster2 | | | |
| --- | --- | --- | --- |
| Variable | Cluster1 (n=184) | Cluster2 (n=142) | p-value |
| Age^a^ |  |  | 0.325 |
| Mean (SD) | 58.75 (10.90) | 59.99 (11.80) |  |
| Pathological diagnosis |  |  | —— |
| Serous | 184 | 142 |  |
| Grade^b^ |  |  | 0.126 |
| G2 | 15 (8.2%) | 19 (13.4%) |  |
| G3 | 169 (91.8%) | 123 (86.6%) |  |
| FIGO stage^b^ |  |  | 0.867 |
| IIIA | 3 (1.6%) | 3 (2.1%) |  |
| IIIB | 8 (4.3%) | 5 (3.5%) |  |
| IIIC | 142 (77.2%) | 114 (80.3%) |  |
| IV | 31 (16.8%) | 20 (14.1%) |  |
| Treatment response^b^ |  |  | 0.091 |
| CR | 97 (52.7%) | 86 (60.6%) |  |
| PR | 26 (14.1%) | 16 (11.3%) |  |
| SD | 8 (4.3%) | 12 (8.5%) |  |
| PD | 13 (7.1%) | 11 (7.7%） |  |
| Unknown | 40 (21.7%) | 17 (12.0%) |  |
| Residual tumor^b^ (post-operation) |  |  | 0.891 |
| <1cm | 118 (64.1%) | 93 (65.5%) |  |
| ≥1cm | 49 (26.6%) | 38 (26.8%) |  |
| Unknown | 17 (9.2%) | 11 (7.7%) |  |
| Survival status^b^ |  |  | 0.527 |
| Live | 65 (35.3%) | 55 (38.7%) |  |
| Dead | 119 (64.7%) | 87 (61.3%) |  |
| Follow-up time (year) |  |  | —— |
| Median (interquartile range) | 2.42 (0.95-4.49) | 3.06 (1.83-4.97) |  |
| * p < 0.05, statistically significant. a Using t-test or ANOVA, P < 0.05 was considered statistically significant. b Using Chi-squared test, P < 0.05 was considered statistically significant.  Abbreviation: HGSOV, High-grade serous ovarian cancers; TCGA, The Cancer Genome Atlas; SD, Standard deviation; G2, Moderately differentiated; G3, Poorly differentiated; FIGO, International Federation of Gynecology and Obstetrics; CR, Complete remission; PR, Partial remission; SD, Stable disease; PD, Progressive disease | | | |

| Table S2. Clinicopathological characteristics of advanced patients with HGSOC between high-risk and low-risk groups | | | |
| --- | --- | --- | --- |
| Variable | Low risk (n=163) | High risk (n=163) | p-value |
| Age^a^ |  |  | 0.238 |
| Mean(SD) | 60.03 (11.26) | 58.55 (11.32) |  |
| Pathological diagnosis |  |  | —— |
| Serous | 163 | 163 |  |
| Grade^b^ |  |  | 0.469 |
| G2 | 19 (11.7%) | 15 (9.2%) |  |
| G3 | 144 (88.3%) | 148 (90.8%) |  |
| FIGO stage^b^ |  |  | 0.855 |
| IIIA | 4 (2.5%) | 2 (1.2%) |  |
| IIIB | 7 (4.3%) | 6 (3.7%) |  |
| IIIC | 127 (77.9%) | 129 (79.1%) |  |
| IV | 25 (15.3%) | 26 (16.0%) |  |
| Treatment response^b^ |  |  | 0.049* |
| CR | 103 (63.2%) | 80 (49.1%) |  |
| PR | 18 (11.0%) | 24 (14.7%) |  |
| SD | 11 (6.7%) | 9 (5.5%) |  |
| PD | 7 (4.3%) | 17 (10.4%） |  |
| Unknown | 24 (14.7%) | 33 (20.2%) |  |
| Residual tumor^b^ (post-operation) |  |  | 0.119 |
| <1cm | 110 (67.5%) | 101 (62.0%) |  |
| ≥1cm | 36 (22.1%) | 51 (31.3%) |  |
| Unknown | 17 (10.4%) | 11 (6.7%) |  |
| Survival status^b^ |  |  | 0.039* |
| Live | 69 (42.3%) | 51 (31.3%) |  |
| Dead | 94 (57.7%) | 112 (68.7%) |  |
| Follow-up time (year) |  |  | —— |
| Median (interquartile range) | 3.31 (1.55-5.26) | 2.42 (1.25-3.95) |  |
| * p < 0.05, statistically significant. a Using t test or ANOVA, P < 0.05 was considered statistically significant. b Using Chi-squared test, P < 0.05 was considered statistically significant.  Abbreviation: HGSOV, High-grade serous ovarian cancers; TCGA, The Cancer Genome Atlas; SD, Standard deviation; G2, Moderately differentiated; G3, Poorly differentiated; FIGO, International Federation of Gynecology and Obstetrics; CR, Complete remission; PR, Partial remission; SD, Stable disease; PD, Progressive disease | | | |
